# Report on Dental Literature and Dental Education

**Published:** 1886-02

**Authors:** W. W. H. Thackston


AMERICAN JOURNAL

OF

DENTAL SCIENCE.

VOL. XIX. THIRD SERIES.—FEBRUARY 1886. No. 10.

ARTICLE I.

### REPORT ON DENTAL LITERATURE AND DENTAL EDUCATION.

BY W. W. H. THACKSTON, M. D., D. D. S.

(Read before the Virginia State Dental Association, October 6th, 1885.)

*Mr. President, and Gentlemen of the State Dental Association:*

In discharging the duty, and, as best I may, fulfilling the trust you have devolved upon me, I take pleasure in reporting a very satisfactory and encouraging condition of our professional literature. Valuable and important contributions have recently been made, and marked and decided advantages accomplished by new and largely original standard works in the several departments of dental science; and new and fresh editions of the older recognized text-books have been brought out, with such corrections, emendations and additions, as were demanded by the present needs and status of our department of the "healing art."

The grand old works of Harris, the father and founder of scientific "American Dentistry," have been ably and faithfully revised, pruned, amended and added to, until they now comprise an encyclopœdia of dental science and knowledge. A new and improved edition of Garretson's "Oral Surgery" brings that masterly work to the front as the embodiment of the latest advances and improvement in our surgical domain. Bond's "Dental Medicine" has been supplemented and succeeded by the more elaborate and comprehensive work of Prof. F. J. S. Gorgas. Kingsley's "Oral Deformities" is a well-nigh exhaustive treatise upon that important part of our art, in which the accomplished author has achieved a world-wide renown. Taft's "Operative Dentistry" and Richardson's "Mechanical Dentistry," Flagg's "Plastics and Plastic Operations," Arthur's "Prophylaxis and Treatment of Dental Decay," Hietsman's "Microscopical Morphology," Essig's "Dental Metallurgy," Seabury and Gilbert's "Vulcanite and Celluloid," and the compilation of Webb's articles on tooth filling, cohesive gold, and the electro magnetic mallet, are all works replete with information and instruction. But alas! it must in truth be said, all are more or less marked and stamped by the errors and imperfections incident to human efforts and human enterprises.

The works and authors I have named are representative of "American dentistry"—of modern and advanced American dentistry—*that* dentistry which, in simple truth, and without vanity, arrogance or egotism, may be said to have led, enlightened and elevated dental science throughout the civilized world.

It is now the fashion, and I may say the folly, in some quarters, to parade and make a sensation—or, as our Western friends would phrase it, "whoop up" the writings, researches and investigations of our transatlantic scientists and authors. Now, sir, while I most cheerfully bear testimony to the worth and value of many foreign workers in our common field, and place a high estimate upon all they have accomplished—nay, more, while I ungrudgingly award them all the fame and distinction to which their talents and learning and labors entitle them; and while I claim to be neither partial on the one hand nor prejudiced on the other, I must be permitted to observe, that with the exception of physiology, microscopical and histological anatomy, and pathology, our brethren abroad have *not* measured up to, equaled, or rivaled the work of our American authors and writers to the "manor born," And I make this declaration fearlessly, and in the face of my frank and cheerful acknowledgement of the high value and great worth of such works as have been produced by Tomes, Beale, Huxley, Nasmyth, Hunter, Fox, Bell, Magitott, Wedl, Lieher and Rotenstien, Kolliker, Kock, Bastian, Pasteur, and others which I have neither time nor space to enumerate.

Many of the old European and American standard works in our department of science, which illustrated talent and genius, and profound study and laborious research, and which were of value and commanding importance in their day and time, have been superseded—are now obsolete—and have been consigned to the "tomb of the Capulets," along with the ephemeral and relatively worthless productions—foreign and domestic—of this latter half of the nineteenth century. In the catalogue I have submitted, I may have failed to include some works and authors of real merit and value, and if so, I shall be most happy to make the correction and the *amende*.

Of our journalistic and periodical literature, I am able to report not only a creditable and gratifying advance, but a supply equal to our growing demands and requirements. All over the land, from Baltimore, the birth-place and cradle of the first dental journal, to San Francisco, on the Pacific; from the Canada line to the Rio Grande, on our southern border, we have monthly or quarterly "journals," teeming with the discourses, inventions, improvements and advances, fresh and fast, as they are being made in all the departments of our calling. The editorial conduct and original contributions to our current literature demonstrate not only improved scholarship, but exhibit a deeper and broader range of thought, a more profound research, and a more laborious and thorough investigation of the subjects and problems that engage our interest and attention as progressive dentists, than has marked any previous era in our professional annals.

Many—very many—of the voluntary papers—the society essays, the committee reports, the transactions and discussions of our annual and monthly meetings, are of a character and quality that reflect dignity and honor upon our profession, and challenge the respect and admiration of our peers and co-workers in all the wide domain of general science.

While we frankly admit that there is in the material and make-up of many of our periodicals much that is amenable to just and sharp criticism, it is nevertheless true, that scattered along through a current volume, you will find articles, essays, and monographs that would honor and embellish the medical and sugical literature of this or any other country—articles and contributions that, in composition and purity of style, would do no discredit to "Blackwood," or the "British Quarterly." We lament the fact that a considerable per centage of our fellow-dentists, though they may be expert and skilful operators, lack the training and qualifications indispensable to finished and ready writing. However, we can yet point to a number, and that number is annually increasing, whose minds have been trained and disciplined, whose style has been cultured and refined, whose pens are dipped in *brain*, and whose productions, in mental grasp and vigor, in purity of taste and beauty of diction, place them alongside the acknowledged representatives of English and American letters.

The editorial revision and typographical execution of several of our leading periodicals and journals deserve our warmest praise and highest commendation; but, unfortunately, others exhibit a melancholy lack of care and attention in these important regards, and so marred, and blurred, and blemished are their pages by false orthography, by incorrect punctuation, by the presence of utterly foreign and irrelevant terms and phrases, as to disgust some of the best writers in our ranks, and to confound and confuse even the professional reader, who is often compelled, as best he can, to *guess* at the real meaning and intent of his author's text. And I here take leave to say, that no one who assumes the duties and responsibilities of editor or publisher, has the moral right to neglect the careful scrutiny and correction of his "proof-sheets," or to travesty or make ridiculous the accepted work of his contributors, by placing that work before the public in a published form that should bring the blush of shame to the cheek of even a "printer's devil." Such neglect and delinquency is an outrage upon correspondents, contributors and subscribers; nay, more, it is a gross and shameful outrage upon a profession which these men claim to represent, and to which they appeal for maintenance and support. I do not wish, in such a report as I am now making, to be personal. I allude to this striking and capital defect in some of our current literature, in no spirit of hypercriticism, and with no disposition to be severely censorious, but in the trust and hope that this blot upon our professional journalism may no longer offend the sensibilities of our writers, or disgust the judgment and taste of our readers; in the trust and hope that our editors and publishers, if incompetent, or wilfully derelict, may have the grace to resign their positions, and transfer their duties to capable and competent successors.

In closing this part of my report, I feel that I shall perform a simple act of justice, and at the same time subserve the welfare of science and promote our own professional interests as dentists, by calling to your attention the high character and acknowledged ability of *The Southern Dental Journal*, published in Atlanta, Ga. This monthly journal especially commends itself to the consideration and esteem of Southern dentists by its energy and enterprise, by the care and painstaking with which it is edited and published, and by the able and accomplished corps of writers and contributors it has engaged to fill and enrich its monthly issues. We sincerely think no Southern dentist can afford to do without this relatively local and truly meritorious monthly.

Upon the subject of "Dental Education," we have but few observations and suggestions to make at this time. The facilities and resources of the profession, so far as relates to training and education, would now seem to be ample and well-nigh complete. We have, as I have already shown, a full line of standard text-books in every division and department of our calling; we have an abundant, a helpful, and ever-fresh and instructive current literature, as I have also shown; we have yet the advantages of primary office instruction by private precepters; we have independent and separate schools and colleges, and we now have dental departments in a number of the universities and medical colleges of the country—all officered and equipped full professors, adjunct and assistant professors, demonstrators, and a corps of clinical lecturers and instructors. We have in these institutions lecture halls, infirmaries, laboratories, and dissecting halls, with all the appointments and paraphernalia required in each. We have to overlook and supervise these schools of learning, trustees, boards of visitors, chancellors, and boards of regents. We have for each school, or "dental department," laws, rules and regulations for its conduct and general government, prescribing the terms and conditions of matriculation, the course of study, and the requirements for graduation and degrees.

Now if these terms and conditions are faithfully and honestly observed; if these laws, rules and regulations are enforced, and fairly carried out and executed by our Chancellors, Regents, Visitors and Faculties. what more can be reasonably asked or desired by our professional students or representative practitioners? The prescribed course of study and training is wide and comprehensive; the facilities and provisions—didactic and demonstrative—abundant and complete; the standard for matriculation and graduation, under the recent *wise* and *timely* action of the Associated Faculties of Dental Colleges, is to be raised, and the lecture terms lengthened, and, I repeat, what more can the profession or the intending and most aspiring student for future membership in the profession, expect or desire? I see nothing to suggest except a faithful and conscientious observance and enforcement of these laws and rules, and the prompt and final abandonment of the mischievous absurdity of matriculating applicants for the lecture terms, who present but the single qualification ot "*cash in hand*," to pay for tickets of admission.

With the sharp competition engendered by our superabundance of professional schools, and dental departments of other schools and universities, I know it is a hard thing for the Deans to turn away an illiterate ignoramus, who presents himself, with his pockets plethoric, and bloated with gold or greenbacks, and it was in anticipation of this and *other* evils, that I long ago placed myself on record as opposed to the multiplication of dental schools and colleges and dental departments in other schools. Years ago, if I may be permitted to quote myself, I told this body of Virginia dentists, and, through the press, the dentists of the entire country, that we were overstocking the market in the line of dental colleges; that the aphorism, "Competition was the life of trade," or the life of anything else, was a fallacy; that outside of given limits and conditions, competition was a blight and mildew and death. "Establish a dental college in every county and at every cross-road; make every fifth or tenth or twentieth man or woman a dentist (for they now take all comers, and make dentists of both sexes) and how long would the colleges or their students and graduates exist? We have at present in operation at least *four times* as many dental schools and colleges as are needed or demanded by any exigency or requirement of our educational interests, and all are making a struggle for patronage, for existence, and some of them for supremacy.

All want classes respectable at least in numbers, and the temptation to receive all applicants who can pay, is hard to withstand. I long ago tried, in my humble way, to quiet and restrain our restless and aspiring embryo teachers and professors, but they were ambitious; some said they were only "enterprising"—"the world moved;" and we have had the dental schools all over the land. Some have already perished, others are maintaining a feeble and sickly existence, and the end promises an illustration of the "Darwinian theory of the survival of the fittest."

But I am happy to say we have good and reliable institutions of learning for our students. We have competent, qualified and faithful instructors who are reasonably well sustained and compensated, under the circumstances; and we expect in the not distant future to see the errors and mistakes which have damaged our educational system work their correction and cure in that sure but slow and costly school of "trial and experience."

In concluding this paper, perhaps already too long, I will, Mr. President, submit one or two observations and suggestions—the first to practicing dentists, the second to teachers and professors, the faculties of our schools and colleges. To the private practitioner I would say, be careful how you encourage a young man to select and adopt dentistry as a profession, for relatively few men have the natural aptitude and endowments essential to success in any department of surgery, and especially "dental surgery." It is an unkindness, nay more, it is an outrage, personal and professional, to accept a student, take his money and encourage him to waste his time and exhaust the energies of his fresh young life in the hopeless attempt to master a science or profession for which he is unfitted by lack of natural talent or lack of preliminary cultivation and training. When, however, you have the opportunity and inclination to accept a student of capacity and promise, teach him faithfully and thoroughly; but as a rule, if you would do him the highest service in your power, send him to the

Infirmary and Laboratory of one of our best colleges. Very few dentists in practice, whatever may be their eapacity to teach, have time to devote to private students.

And secondly, I would say to the faculties of our schools, teach thoroughly every department of your curriculum, raise and keep high your standard of graduation; teach not only dental science, but teach a *moral* and *ethical code* that will enable you to send from your halls not only qualified dentists, but truthful, honorable and accomplished gentlemen; for to such alone do we desire to commit the future destiny of "American Dentistry."—*Southern Dental Journal.*

---

ARTICLE II.

